# Pseudo-Tuberculosis

**Published:** 1901-05-25

**Authors:** 


### Hospital Clinics and Medical Progress.

### PSEUDO-TUBERCULOSIS.

The annual report of the medical officer of the Local Government Board, which has just been issued, contains much that is interesting to pathologists as well as to those engaged in public health work. Among the auxiliary scientific investigations undertaken by direction of the department is an important one by Dr. Klein on pseudo-tuberculosis, to which Mr. Power draws special attention. In making use of such a vague and in many respects objectionable term as pseudo-tuberculosis, it is desirable in the first instance to define what one means by it. Many pathological conditions met with in the lower animals, although in appearance they simulate tubercle, yet are not associated with tubercle bacilli, and such conditions have occasionally been described as "pseudo-tuberculous." The term "pseudo-tuberculosis," however, has also come to be used in a more restricted manner to denote a single disease, pathologically akin to tuberculosis, occurring in certain lower animals under natural conditions, transmissible from animal to animal, and caused by the life processes of a specific bacillus, which, however, is quite distinct from the bacillus tuberculosis. The bacillus in question was first isolated by Pfeiffer, and has been named the "bacillus pseudo-tuberculosis." Thus, just as when we speak of tuberculosis, whatever its form or manifestation, we mean a disease set up by the bacillus tuberculosis; when we speak of pseudo-tuberculosis we mean, according to Dr. Klein, a malady set up by the bacillus pseudo-tuberculosis. Evidently it is a bad name, but there it is.

Dealing with the subject as so defined Dr. Klein has investigated and described the clinical history and the post-mortem appearances of numerous animals in which this disease had been set up. The animals used in the experiments were mostly guinea-pigs, rabbits, and mice, and in them it was found that the disease was more rapid than true tuberculosis, and that histologically the "giant cell" formation so common in tubercle was almost entirely absent, and each disease was of course accompanied by its characteristic bacillus. But in nearly all other respects the pathological similarity between pseudo-tuberculosis and tuberculosis was strikingly close. Moreover, it deserves notice that this similarity was in no cases more marked than when the disease had been artificially induced by feeding. Dr. Klein has shown that the pseudo-bacillus is very widely distributed. Thus he has detected it in samples of unfiltered Thames and sea-water, in sewage, and in certain sewage effluents. The most striking discovery, however, has regard to the frequency of its presence in milk. Two out of five samples of milk purchased at London shops, and six out of 97 samples of milk delivered at London railway stations direct from dairy farms, produced pseudo-tuberculosis on inoculation into guinea-pigs. The importance of this discovery is very great in view of the frequency with which inoculation experiments are performed with the object of ascertaining whether or no certain samples of milk are tainted with tuberculosis; for it is clear that the production in the inoculated animal of lesions resembling tubercle must not be taken as proof of the presence of tuberculous infection unless these lesions in turn are shown to contain the bacillus tuberculosis. It has been shown that when the two forms of microbe are inoculated together, or within a short time of one another, the two diseases are capable of progressing simultaneously, although not at equal rates, in one and the same animal. Hence, in such an animal lesions which seemingly form part of a single pathological process may be found associated, some with tubercle bacilli alone, others with bacilli of pseudo-tuberculosis alone, and elsewhere with a mixed infection of both these microbes. This has a bearing upon the possibility of the disease affecting man. Dr. Klein says that the disease may be readily communicated to the ape, and that other observers have claimed that some nodular deposits in the human subject, apparently tuberculous, are really pseudo-tuberculous. In view of the facts shown, Dr. Klein, in the cautious words appropriate to a blue book, thinks "it is not too much to say that the presence of the bacillus of pseudo-tuberculosis in milk may probably play a part in causing pseudo-tuberbulous disease in the human subject."

It may be well while dealing with this matter to refer to the discussion on the subject which took place at the Pathological Society a couple of years ago, when a very strong opinion was expressed against the continued use of the name "pseudo-tuberculosis." As Professor Sims Woodhead said in introducing the subject, "the number of conditions classified within this term are so numerous that 'pseudo-tuberculosis' is no longer of the slightest value as a term in classification."

After describing a variety of conditions which had been included under the term pseudo-tuberculosis, on account of the apparent similarity of the lesions produced, he went on to say that in comparatively recent times we had passed on from the anatomical pseudo-tuberculosis to a pseudo-tubercle bacillus, a factor which he said promised, unless taken firmly in hand, and in time, to render the question still more complicated. The complication so prophesied has now arisen in full force, and in view of the almost unanimous feeling expressed by pathologists against the continuance of the use of the term pseudo-tuberculosis it seems somewhat of a pity that Dr. Klein should give official sanction to its employment in the sense in which he uses it. As a temporary bridge over a pathological gap it was excusable, and even now is convenient, to group under the term pseudo-tuberculosis a considerable number of conditions which, although confessedly not tubercle are very like it. There is, however, but little excuse, and certainly no convenience in picking out from the crowd one of these conditions and dubbing it pseudo-tuberculosis and its bacillus the bacillus pseudo-tuberculosis. So long as it is clearly understood that the term merely means any process which imitates the anatomical lesions produced by the B. tuberculosis its use is convenient enough, but that is exactly the meaning which Dr. Klein will not allow it to have. It is to be confessed, however, that it is not easy to provide a satisfactory substitute for this unfortunate word.

